# 1 h Postload Glycemia Is Associated with Low Endogenous Secretory Receptor for Advanced Glycation End Product Levels and Early Markers of Cardiovascular Disease

**DOI:** 10.3390/cells8080910

**Published:** 2019-08-16

**Authors:** Antonino Di Pino, Francesca Urbano, Roberto Scicali, Stefania Di Mauro, Agnese Filippello, Alessandra Scamporrino, Salvatore Piro, Francesco Purrello, Agata Maria Rabuazzo

**Affiliations:** Department of Clinical and Experimental Medicine, University of Catania, 95122 Catania, Italy

**Keywords:** cardiovascular risk, 1 h postload glycemia, soluble receptor for advanced glycation end products, arterial stiffness, intima–media thickness

## Abstract

We investigated the correlation of the soluble receptor for advanced glycation end products (sRAGE) and endogenous secretory RAGE (esRAGE) with markers of cardiovascular disease in subjects with normal glucose tolerance (NGT) and 1 h postload glucose ≥155 mg/dL after an oral glucose tolerance test. We stratified 282 subjects without a previous diagnosis of diabetes into three groups: 123 controls (NGT and 1 h postload glycemia <155 mg/dL), 84 NGT and 1 h postload glycemia ≥155 mg/dL (NGT 1 h high), and 75 subjects with impaired fasting glucose and/or impaired glucose tolerance (IFG/IGT). NGT 1 h high subjects exhibited lower esRAGE (0.36 ± 0.18 vs. 0.4 5 ± 0.2, *p* < 0.05) and higher S100A12 levels than controls (5684 (3193.2–8295.6) vs. 3960.1 (2101.8–7419), *p* < 0.05). Furthermore, they showed an increased pulse wave velocity (PWV) and intima–media thickness (IMT). No differences were found between the NGT 1 h high group and the IFG/IGT group regarding cardiometabolic profiles. After multiple regression analyses, esRAGE was associated with glycated hemoglobin (HbA_1c_) and high-sensitivity C-reactive protein (hs-CRP). Age, HbA_1c_, and esRAGE were the determinants of IMT, whereas S100A12 and systolic pressure were the determinants of PWV. The NGT 1 h high group exhibited low esRAGE levels and an altered cardiometabolic profile. HbA_1c_, S100A12, and hs-CRP were associated with these alterations. In conclusion, subjects with NGT are not a homogeneous population, and they present different cardiovascular and glycometabolic risks.

## 1. Introduction

Prediabetes, a common disorder of glucose homeostasis, is prevalent in the general population, and affected subjects are at high risk of progression to overt diabetes and cardiovascular disease [1,2]. As such, prediabetes typically represents three groups of individuals: those with impaired fasting glucose (IFG), those with impaired glucose tolerance (IGT), and those with a glycated hemoglobin (HbA_1c_) between 5.7% and 6.4% (39–46 mmol/mol) [3]. Several clinical trials have demonstrated that lifestyle intervention and pharmacological therapy in high-risk individuals could reduce the incidence of type 2 diabetes and prevent or delay the cardiovascular complications associated with both type 2 diabetes and prediabetes itself [4,5,6]. Thus, reliable models for identification of individuals at high risk for future type 2 diabetes are essential and have important clinical implications for intervention programs [7].

An increasing body of evidence has suggested that a plasma glucose concentration of at least 155 mg/dL (8.6 mmol/L) at 1 h during an oral glucose tolerance test (OGTT) can identify adults at increased risk for future development of type 2 diabetes among those who have normal glucose tolerance (NGT 1 h high) [8,9,10]. Furthermore, it has been demonstrated that subjects with NGT 1 h high showed an unfavorable cardiometabolic profile similar to that observed in individuals with IGT. In particular, they exhibited an atherogenic lipid pattern and an early alteration of early markers of vascular damage [11,12,13].

To explain the association between hyperglycemia and vascular complications in disorders of glucose homeostasis, several studies have emphasized the role of advanced glycation end products (AGEs) and their cell surface receptors (RAGEs). The interaction between RAGE and its ligands (AGEs and other molecules, such as S100A12) effectively modulates several steps of atherogenesis, triggering an inflammatory-proliferative process and critically contributing to the propagation of vascular perturbation, mainly in diabetes [14]. RAGE has a secretory isoform, which is termed soluble RAGE (sRAGE). sRAGE is primarily formed by the proteolytic cleavage of membrane-bound RAGE and secondarily by a secreted, non-membrane-bound form of the receptor resulting from alternative splicing of the RAGE gene, which is known as endogenously secreted RAGE (esRAGE). esRAGE may contribute to the removal/neutralization of circulating ligands, thus functioning as a decoy by competing with cell surface RAGE for ligand binding [15].

sRAGE has been recently associated with a greater risk of cardiovascular complications. Several studies have demonstrated an inverse cross-sectional association between sRAGE plasma levels and coronary heart disease or atherosclerosis in nondiabetic men [16]. Prospective studies have shown that low levels of sRAGE predict cardiovascular mortality in diabetic and nondiabetic subjects [17]. Recently, our group reported that patients with HbA_1c_ prediabetes showed lower esRAGE plasma levels than controls, and these levels were independently related with early markers of cardiovascular disease [18].

To date, no information is available regarding the role of the AGE/RAGE axis and circulating esRAGE levels in NGT 1 h high subjects and their possible link with vascular damage in this population.

In this study, we measured sRAGE and esRAGE levels and examined their associations with other proinflammatory factors and early markers of atherosclerosis in NGT subjects with high (≥155 mg/dL) 1 h postload plasma glucose.

## 2. Materials and Methods

### 2.1. Study Design and Participants

This cross-sectional study was conducted on 282 participants with no previous diagnosis of diabetes who attended our University Hospital for diabetes and cardiovascular risk evaluation. The inclusion criterion was an age between 30 and 65 years. All patients were Caucasian and underwent a screening test, which included a physical examination and review of their clinical history, smoking status, and alcohol consumption. Body weight and height were measured, and body mass index (BMI) was calculated as weight (kg)/[height (m)]^2^. Waist circumference (WC) was measured in a standing position at the level of the umbilicus. Blood pressure (BP) was measured with a calibrated sphygmomanometer after the subject had rested in the supine position for 10 min. After an overnight fasting, venous blood samples were obtained for the measurement of biochemical parameters, and 75 g OGTT was administered as previously described [19,20]. The exclusion criteria were as follows: a previous history of diabetes, a previous history of overt cardiovascular events (stroke, ischemic heart disease, chronic obstructive peripheral arteriopathy, or heart failure), anemia or hemoglobinopathies, the use of medications known to affect glucose metabolism, clinical evidence of liver or renal disease, chronic diseases, and/or recent history of acute illness, malignant disease, and drug or alcohol abuse.

### 2.2. Biochemical Analyses

Plasma glucose, total cholesterol, triglycerides, high-density lipoprotein (HDL) cholesterol, and high-sensitivity C-reactive protein (hs-CRP) were measured using available enzymatic methods, as previously described [21]. Low-density lipoprotein (LDL) cholesterol concentrations were estimated using the Friedewald formula. All subjects underwent a 75 g OGTT with sampling for glucose and insulin. Glucose tolerance status was defined on the basis of OGTT according to the American Diabetes Association (ADA) recommendations [3].

To quantify the plasma concentration of sRAGE (Human sRAGE ELISA; Biovendor), esRAGE (B-Bridge esRAGE ELISA Kit), carboxymethyl-lysine (CML), (CircuLex, ELISA Kit for CML-Nε_(Carboxymethyl)lysine), and S100A12 (Cloud-Clone Corp., Houston, TX, USA, ELISA Kit for S1000A12), fasting blood samples were centrifuged and stored at −80 °C. HbA_1c_ was measured via HPLC using a National Glycohemoglobin Standardization Program and was standardized to the Diabetes Control and Complications Trial assay reference [22].

### 2.3. Carotid Ultrasound Examination

Ultrasound scans were performed using a high-resolution B-mode ultrasound system (MyLab 50 XVision; Esaote Biomedica SpA, Florence, Italy) equipped with a 7.5 MHz linear array transducer. To exclude interobserver variability, all ultrasound examinations were performed by a single physician who was blinded to the clinical and laboratory characteristics of the participants. The subjects were examined in the supine position. Longitudinal images from the angle with the best visibility were displayed bilaterally for the common carotid artery. Scans were performed and measurements were conducted at a total of six plaque-free sites 1 cm proximal to the carotid bulb. The obtained values were averaged and are presented as the mean of the intima–media thickness (IMT) of the common carotid artery. Plaques, defined as a clearly isolated focal thickening of the intima–media layer with a thickness of 1.4 mm, were not observed in any individuals. All measurements were obtained in diastole, assessed as the phase in which the lumen diameter is at its smallest and the IMT is at its largest.

### 2.4. Arterial Stiffness Evaluation

#### 2.4.1. Pulse Wave Velocity

The SphygmoCor CVMS (AtCor Medical, Sydney, Australia) system was used for the determination of the pulse wave velocity (PWV), as already described [23]. This system uses a tonometer and two different pressure waves obtained at the common carotid artery (proximal recording site) and at the femoral artery (distal recording site). The distance between the recording sites and suprasternal notch was measured using a tape measure. An electrocardiogram was used to determine the start of the pulse wave. The PWV was determined as the difference in travel time of the pulse wave between the two different recording sites and the heart, divided by the travel distance of the pulse waveform. The PWV was calculated on the mean of 10 consecutive pressure waveforms to cover a complete respiratory cycle.

#### 2.4.2. Pulse Wave Analysis

The SphygmoCor CVMS (AtCor Medical, Sydney, Australia) system was used for the determination of the augmentation pressure (Aug P), augmentation index (Aug I), and subendocardial viability ratio (SEVR). All measurements were made from the right radial artery by applanation tonometry using a Millar tonometer (SPC-301; Millar Instruments, Houston, TX, USA). The measurements were performed by a single investigator with the subject in the supine position, as previously described [24].

### 2.5. Statistical Analyses

The sample size was calculated based on previous studies examining esRAGE differences among patients with altered glycemic homeostasis and control subjects; the level of significance (α) was set to 5% and power (1-β) set to 80%. Statistical comparisons of the clinical and biomedical parameters were performed using Stat View 6.0 for Windows. The data are presented as the means ± SD or median (interquartile range, IQR). A *p*-value less than 0.05 was considered statistically significant. The estimated sample size was 70 patients for each group.

The distributional characteristics of each variable, including normality, were assessed by the Kolmogorov–Smirnov test. The statistical analyses were performed with the unpaired Student’s *t* test and ANOVA for continuous variables and the χ^2^ test for noncontinuous variables. When necessary, numerical variables were logarithmically transformed for statistical analysis to reduce skewness.

To identify variables independently associated with variations of esRAGE, IMT, and PWV in NGT 1 h high subjects, we performed three multivariate regression models. The first model included several clinical characteristics (age, sex, BMI, smoking status, systolic and diastolic BP, HDL cholesterol, and LDL cholesterol). Subsequently, variables reaching significance were inserted in a second model that included variables related with glucose homeostasis ((HbA_1c_, fasting glycemia, 1 h post load glycemia, 2 h post load glycemia, homeostasis model assessment of insulin resistance (HOMA-IR)). Subsequently, variables reaching significance were inserted in a third model that included glycation and inflammatory markers (sRAGE, esRAGE, CML, hs-CRP, S100A12). The variance inflation factor was used to check for the problem of multicollinearity in multiple regression analysis.

All subjects gave their informed consent for inclusion before they participated in the study. The study was conducted in accordance with the Declaration of Helsinki and the protocol was approved by the Ethics Committee Catania 2 (24/CE/2012).

## 3. Results

In total, 282 subjects participated in the study. The study population was divided into three groups based on fasting and 1 and 2 h postload glycemia: 123 controls (NGT and 1 h postload glycemia <155 mg/dL), 84 NGT and 1 h postload glycemia ≥155 mg/dL (NGT 1 h high), and 75 subjects with impaired fasting glucose and/or impaired glucose tolerance (IFG/IGT).

The clinical and biochemical characteristics of the study population are presented in Table 1. NGT 1 h high subjects were older than the controls but were similar with regard to BMI, WC, total cholesterol, systolic BP, and diastolic BP. Subjects with IFG/IGT were similar to NGT 1 h high with respect to anthropometric and metabolic characteristics except for age, fasting glucose, 1 and 2 h postload glucose, HbA_1c_, and HOMA-IR (Table 1).

**Figure 1:**
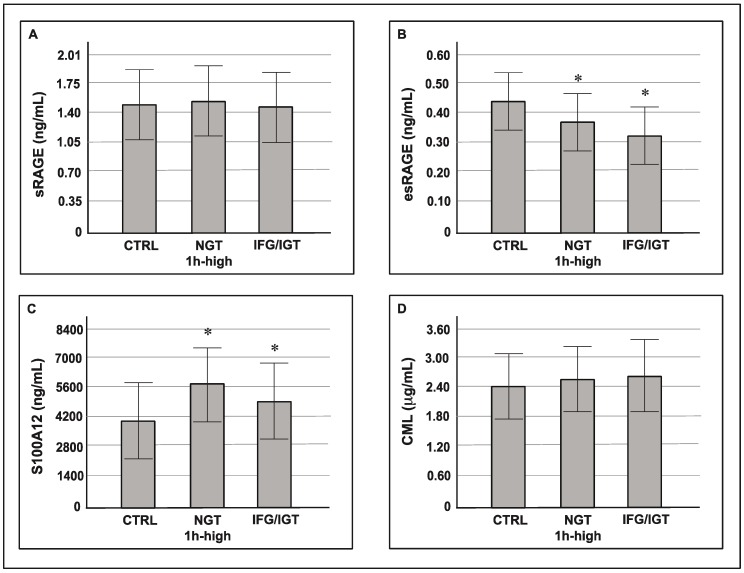
Circulating levels of soluble receptor for advanced glycation end-products (sRAGE) (**A**), endogenous secretory RAGE (esRAGE) (**B**), S100A12 (**C**), and carboxymethyl-lysine (CML) (**D**) (mean ± SD) according to glucose tolerance and 1 h postload plasma glucose. * *p* < 0.05 vs. CTRL.

The circulating plasma levels of esRAGE were lower in the NGT 1 h high group compared with the control group (0.36 ± 0.1 vs. 0.45 ± 0.12, *p* < 0.05). Furthermore, subjects with 1 h ≥155 mg/dL showed higher S100A12 and hs-CRP serum levels (5684 (3193.2–8295.6) vs. 3960.1 (2101.8–7419) *p* < 0.05 and 0.24 (0.12–0.43) vs. 0.15 (0.1–0.25), *p* < 0.05, respectively). sRAGE and CML plasma levels were similar among the three groups. There were no differences between the NGT 1 h high group and the IFG/IGT group with respect to inflammatory markers (sRAGE, esRAGE, S100A12, CML, and hs-CRP) (Figure 1).

The NGT 1 h high group showed alterations of early markers of vascular damage compared with the control group, i.e., higher IMT (0.78 (0.68–0.83) vs. 0.69 (0.56–0.71), *p* < 0.05), PWV (7.7 ± 1.4 vs. 7.22 ± 1.6, *p* < 0.05), Aug P (11.6 ± 6.1 vs. 9.2 ± 6.5, *p* < 0.05), Aug I (28.6 ± 11.7 vs. 25.2 ± 12, *p* < 0.05). Moreover, IMT and PWV were increased in the IFG/IGT group compared with subjects with 1 h ≥155 mg/dL (0.83 (0.71–0.92) vs. 0.78 (0.68–0.83), *p* < 0.05; 8.2 ± 1.6 vs. 7.7 ± 1.4, *p* < 0.05). No differences were found in the SEVR between the three groups (Table 2).

To identify variables independently associated with variations of esRAGE, IMT, and PWV in the NGT 1 h high group, we performed three multivariate regression models (see Statistical Analysis). esRAGE exhibited a significant correlation with HbA_1c_ and systolic BP in the first and second models. In the third model, the variables that remained significantly associated with esRAGE were HbA_1c_ (*p* = 0.05) and hs-CRP (*p* = 0.05) (Table 3).

IMT was independently associated with age and HbA_1c_ in the first and second models. In the third model, IMT showed a significant association with age (*p* = 0.001), HbA_1c_ (*p* = 0.04), and esRAGE levels (*p* = 0.005). PWV was associated with age and systolic BP in the first and second models; the only variable associated with PWV in the third model was S100A12 plasma levels (*p* = 0.04) (Table 3).

## 4. Discussion

In this study, we measured sRAGE, esRAGE, and other markers of inflammation in subjects with high 1 h postload glycemia and examined their association with early markers of cardiovascular damage. Data from other studies have underlined a broad range of metabolic abnormalities in subjects with high 1 h postload glycemia, such as an increased whole blood viscosity, liver enzymes and uric acid, and reduced vitamin D plasma levels [25,26,27,28]. However, none of these studies explored the AGE/RAGE axis in this population.

We found that the NGT 1 h high group showed lower esRAGE plasma levels compared with the control group. Furthermore, these levels were independently associated with HbA_1c_ in the multiple regression analysis. These results are in line with other studies investigating inflammatory profiles in patients with high 1 h postload glucose. Fiorentino et al. provided evidence that NGT 1 h high subjects had an unfavorable inflammatory profile, as measured by the inflammatory score, compared with NGT 1 h low individuals in a study conducted on 1099 Caucasian subjects. Similar to our study, NGT 1 h high subjects exhibited a clustering of inflammatory markers similar to that observed in patients with IGT [29]. The association between the RAGE axis and several alterations of glucose homeostasis has been explored in previous studies. Our group showed reduced levels of esRAGE in subjects with NGT and HbA_1c_ between 5.7% and 6.4% [18,30]. Koyama et al. found that esRAGE was significantly and inversely correlated with HbA_1c_ and components of the metabolic syndrome in subjects with and without type 2 diabetes. Furthermore, Katakami et al. reported an inverse and significant association between esRAGE and HbA_1c_ in patients with type 2 diabetes mellitus [31,32].

In this study, we showed an alteration of early markers of cardiovascular risk (IMT and arterial stiffness) in subjects with high 1 h postload glycemia. Furthermore, HbA_1c_ was associated with increased IMT independently from atherosclerotic risk factors and glucose homeostasis parameters. The risk of CVD is increased before glycemia reaches diabetic levels; however, which among the glucose homeostasis parameters (fasting plasma glucose, 1 h postload glucose, 2 h postload glucose, and/or HbA_1c_) could be a better predictor of CVD remains unclear [16]. Contrary to HbA_1c_, 1 h postload glucose determination is not currently recommended by the American Diabetes Association for identifying individuals at high-risk for type 2 diabetes. However, previous studies reporting the association between cardiovascular risk and 1 h postload glycemia have underlined the importance of obtaining intermediate plasma glucose levels during the oral glucose tolerance test [11,33]. A two-step approach, using an OGTT in addition to HbA_1c_ measurement, has been proposed by several authors. They have reported that 1 h postload glucose determination may be helpful in identifying a subset of individuals within HbA_1c_-defined prediabetes at higher risk for cardiovascular disease and hepatic steatosis [34]. This method could appear inconvenient or expensive; however, the information provided by this approach may allow the identification of individuals at greater risk, who will mostly benefit from lifestyle or pharmacological interventions. Future studies are needed to verify the cost/effectiveness of this type of method.

This study has several limitations. First, the OGTT was performed once; thus, although it reflects real clinical practice, intraindividual variation in plasma glucose levels cannot be taken into account. Second, the cross-sectional design of the study reflects only an association with the most prevalent cardiometabolic variables and precludes us from drawing any conclusion about causal relationships between dysglycemic conditions assessed by 1 h postload plasma glucose determination and clinically relevant cardiovascular disease. Third, this study is based on outpatients recruited at a referral university hospital, representing individuals at increased risk for cardiometabolic disease, ir thereby limiting thegeneralizability to the general population.

In conclusion, subjects with high 1 h postload glycemia exhibit low esRAGE plasma levels and altered markers of cardiovascular disease. Based on these data, subjects with NGT are not a homogeneous population of patients, and they present different cardiovascular and glycometabolic risks.

## Figures and Tables

**Table 1:** Clinical and metabolic characteristics of the study population according to 1 h postload plasma glucose and glucose tolerance.

	NGT 1 h <155 mg/dL (*n* = 123)	NGT 1 h ≥155 mg/dL (*n* = 84)	IFG/IGT (*n* = 75)
**Age (year)**	45 ± 10.2	47.5 ± 10 *	49.5 ± 7.8 *#
**BMI (Kg/m^2^)**	29.5 ± 5	29.2 ± 4	29.8 ± 6.1
**Waist circumference (cm)**	99.9 ± 11.7	100 ± 10.8	98.3 ± 9.6
**Fasting glucose (mg/dL)**	86.4 ± 8.6	90.5 ± 8.6 *	99.4 ± 14 *#
**1 h postload glucose (mg/dL)**	121.3 ± 21.1	176 ± 22.4 *	187.6 ± 35.4
**2 h postload glucose (mg/dL)**	105.3 ± 22	119 ± 21.4 *	168.4 ± 22.4
**HbA_1c_ (%)**	5.6 ± 0.3	5.8 ± 0.38 *	6.0 ± 0.31
**Fasting insulin (μU/mL)**	7.2 ± 3.6	9 ± 6.5	10.8 ± 5.6
**Total cholesterol (mg/dL)**	192.2 ± 36.7	197.4 ± 42.1	199.4 ± 40.4
**HDL cholesterol (mg/dL)**	49.1 ± 11.4	44 ± 12.3 *	43.1 ± 11.8 *
**Triglycerides (mg/dL)**	86 (66–122)	100 (76–137)	125.5 (85–173)
**LDL cholesterol (mg/dL)**	124.1 ± 32.4	128.4 ± 41.1 *	125.2 ± 33.5
**Systolic BP (mmHg)**	118.2 ± 15.6	121 ± 13.5	123.9 ± 14.2 *
**Diastolic BP (mmHg)**	73.2 ± 11	74.4 ± 10.6	75.1 ± 10.4
**HOMA-IR**	1.55 ± 0.84	2.09 ± 1.55 *	2.3 ± 1.5 *#
**Hypertension**	16%	26%	31%
**ACE inhibitors/ARB**	50%	50%	52%
**Calcium channel blockers**	20%	22%	22%
**Beta blockers**	5%	9%	8%
**Thiazide diuretics**	15%	5%	8%
**No therapy**	10%	14%	12%
**Statin therapy**	18%	20%	22%
**Active smokers**	30%	26%	34%
**Sex (M/F)**	41/82	41/42	35/40

Data are presented as mean ± SD or median (interquartile range, IQR). NGT, normotolerant and normal fasting glucose; IFG/IGT, impaired fasting glucose and impaired glucose tolerance; BMI, body mass index; HbA_1c_, glycated hemoglobin; HDL, high-density lipoprotein; LDL, low-density lipoprotein; BP, blood pressure; HOMA-IR, homeostasis model assessment of insulin resistance; ACE, angiotensin-converting enzyme; ARB, angiotensin receptor blockers. Smoking was quantified (number of cigarettes and years smoked) and smoking status was classified in active and nonsmokers. Hypertension was defined as systolic blood pressure ≥135 mmHg or diastolic blood pressure ≥85 mmHg or taking any hypertension medications. * *p* < 0.05 vs. NGT 1 h <155 mg/dL; #*p* < 0.05 vs. NGT 1 h ≥155 mg/dL.

**Table 2:** Early markers of cardiovascular damage according to 1 h postload plasma glucose and glucose tolerance.

	NGT 1 h <155 mg/dL (*n* = 123)	NGT 1 h ≥155 mg/dL (*n* = 84)	IFG/IGT (*n* = 75)
**IMT (mm)**	0.69 (0.56–0.71)	0.78 (0.68–0.83) *	0.83 (0.71–0.92) *#
**PWV (cm/sec)**	7.22 ± 1.6	7.7 ± 1.4 *	8.2 ± 1.6 *#
**Aug P (mmHg)**	9.2 ± 6.5	11.6 ± 6.1 *	12.7 ± 6.1 *
**Aug I (%)**	25.2 ± 12	28.6 ± 11.7 *	30.6 ± 11.7 *
**SEVR (%)**	162 ± 27.7	158 ± 30.8	158.2 ± 32.3

Data are presented as mean ± SD or median (IQR). NGT, normotolerant and normal fasting glucose; IFG/IGT, impaired fasting glucose and impaired glucose tolerance; IMT, intima–media thickness; PWV, pulse wave velocity; Aug P, augmentation pressure; Aug I, augmentation index; SEVR, subendocardial viability ratio. *p < 0.05 vs. NGT 1 h < 155 mg/dL; #*p* < 0.05 vs. NGT 1 h ≥155 mg/dL.

**Table 3:** Multiple regression analysis evaluating esRAGE, IMT, and PWV as dependent variables.

	Coefficient β	*p*-Value
**esRAGE**		
Model 1 *		
Systolic BP	0.29	0.03
Model 2 **		
HbA_1c_	−0.18	0.01
Model 3 ***		
HbA_1c_	−0.27	0.05
hs-CRP	−0.35	0.04
**IMT**		
Model 1 *		
age	0.52	0.001
Model 2 **		
age	0.51	0.001
HbA_1c_	0.2	0.05
Model 3 ***		
age	0.51	0.001
HbA_1c_	0.35	0.04
esRAGE	−0.21	0.005
**PWV**		
Model 1 *		
age	0.25	0.006
Systolic BP	2.4	0.01
HDL	−2.5	0.01
Model 2 **		
age	0.16	0.04
Systolic BP	0.31	0.001
Model 3 ***		
S100A12	0.31	0.04

*Model 1 adjusted for age, sex, body mass index (BMI), systolic BP, diastolic BP, LDL cholesterol, and HDL cholesterol. **Model 2 adjusted for HbA_1c_, fasting glycemia, 1 and 2 h postload glycemia, and HOMA-IR. ***Model 3 adjusted for esRAGE, hs-CRP, S100A12, and CML.
